# Assessing medical students' attitudes towards learning communication skills – which components of attitudes do we measure?

**DOI:** 10.1186/1472-6920-7-4

**Published:** 2007-03-30

**Authors:** Tor Anvik, Tore Gude, Hilde Grimstad, Anders Baerheim, Ole B Fasmer, Per Hjortdahl, Are Holen, Terje Risberg, Per Vaglum

**Affiliations:** 1 Institute of Community Medicine, Faculty of Medicine, N-9037 University of Tromsø, Norway; 2 Department of Behavioural Sciences in Medicine, Faculty of Medicine, N-0317 University of Oslo, Norway; 3 Department of Public Health and General Practice, Norwegian University of Science and Technology, N-7489 Trondheim, Norway; 4 Department of Public Health and Primary Health Care, N-5020 University of Bergen, Norway; 5 Department of Clinical Medicine, Faculty of Medicine, N-5020 University of Bergen, Norway; 6 Institute of General Practice and Community Medicine, Faculty of Medicine, N-0317 University of Oslo, Norway; 7 Faculty of Medicine, Norwegian University of Science and Technology, N-7489 Trondheim, Norway; 8 Institute of Clinical Medicine, Faculty of Medicine, N-9037 University of Tromsø, Norway

## Abstract

**Background:**

The Communication Skills Attitudes Scale (CSAS) created by Rees, Sheard and Davies and published in 2002 has been a widely used instrument for measuring medical students' attitudes towards learning communication skills. Earlier studies have shown that the CSAS mainly tests two dimensions of attitudes towards communication; positive attitudes (PAS) and negative attitudes (NAS). The objectives of our study are to explore the attitudes of Norwegian medical students towards learning communication skills, and to compare our findings with reports from other countries.

**Methods:**

The CSAS questionnaire was mailed simultaneously to all students (n = 3055) of the four medical schools in Norway in the spring of 2003. Response from 1833 students (60.0%) were analysed by use of SPSS ver.12.

**Results:**

A Principal component analysis yielded findings that differ in many respects from those of earlier papers. We found the CSAS to measure three factors. The first factor describes students' feelings about the way communication skills are taught, whereas the second factor describes more fundamental attitudes and values connected to the importance of having communication skills for doctors. The third factor explores whether students feel that good communication skills may help them respecting patients and colleagues.

**Conclusion:**

Our findings indicate that in this sample the CSAS measures broader aspects of attitudes towards learning communication skills than the formerly described two-factor model with PAS and NAS. This may turn out to be helpful for monitoring the effect of different teaching strategies on students' attitudes during medical school.

## Background

Medical students' attitudes towards doctor-patient communication have for long been a concern among medical teachers, curriculum planners and policy makers [1,2] and have been addressed in many studies.

Kaufmann [3] constructed the Attitudes Towards Medical Communication Scale with 41 items and used it in a cross-sectional study on 203 students in their first, second and fourth year respectively. This study, which was published in 2001, showed that female students had more positive attitudes than male students, and that first and second year students had more positive attitudes than fourth year students.

In 2001 de Valck [4] presented a questionnaire measuring students' attitudes towards full disclosure versus non-disclosure in breaking bad news. Following one cohort of students for three years (53 students responded in all three years) they found that students became more in favour of non-disclosure as they progressed through medical school.

In 2002 Rees, Sheard and Davies [5] published the Communication Skills Attitudes Scale (CSAS), which measures students' attitudes towards learning communication skills during medical school. This scale has until spring 2006 been used and validated in three different studies in the UK involving from 216 to 490 students [6-8] and one involving 123 students in Nepal [9]. Although mostly cross-sectional, these studies report that female students have more positive attitudes than male, and that students early in medical school have more positive attitudes than students later in medical school. In addition, having recently attended communication skills teaching tends to predict less positive attitudes towards learning such skills.

In 2004 Liddell and Davidson [10] published the use of a questionnaire measuring medical students' attitudes towards five groups of consultation skills, one of which was communication skills. They performed a cross-sectional study of three consecutive classes of 357 final year students before and after attachments in general practice and a Consulting Skills Program. After the program, attitudes towards communication skills were more positive.

Attitudes involve evaluations by which we attach good or bad qualities to a topic or an organisation or a person. Attitudes drive behaviour. If we can change a person's attitude we may change his or her behaviour [11]. Attitudes have three main components: affective (the way we feel), cognitive (the way we think) and behavioural (the way we act) towards a particular entity [11]. Affective attitudes reflect emotional reactions and may change after repeated exposure to situations involving the goal for the attitude. Cognitive components of attitudes are believed to be more fundamental and constant over time and more closely connected to basic values [12]. Cognitive attitudes are difficult to influence but may change when new knowledge is presented; provided the knowledge is convincing and the presenter is credible [13]. Behavioural attitudes are manifestations of underlying cognitive and affective attitudes. There is evidence that changing behaviour by training new ways of acting in professional situations may influence the more fundamental aspects of attitudes without targeting them directly [14]. There is need for assessment tools enabling teachers and curriculum planners to monitor changes in specific components of attitudes among students during medical school. The use of such tools may also facilitate comparisons between different medical schools. Such comparisons are important because differences in attitudes may to some extent be linked to differences in teaching methods and/or curriculum designs, thereby helping medical educators in finding new ways of improving and refining teaching in medical schools [15].

The aims of our study are to explore the attitudes among all Norwegian medical students towards learning communication skills, and to compare our findings with reports from other countries.

## Methods

### Participants

There are four medical schools in Norway (4.5 million inhabitants), and all of them have curricula lasting six years. Uptake into medical school is based on identical national criteria from high-school exams. Three of the schools have integrated curricula, with parallell pre-clinical and clinical training, and two are PBL-based. Communication skills are taught in all four schools but in different ways and at different stages in school. The particulars of each school are presented in more detail in [16] and [17].

In May 2003 we sent letters by post to the 3055 medical students who had registered their addresses at the universities of Tromsø (A), Trondheim (B), Bergen (C) and Oslo (D). The letters enclosed a description of the study and the Communication Skills Attitude Scale (CSAS) questionnaire. Additional questionnaires asked for background information such as gender, age, previous education and job experience before and during medical school and tested self-reported communication skills, perceived medical school stress and knowledge of communication skills. We posted a reminder to all students after three weeks and again by e-mail after six weeks. The overall response after two reminders was 1833 out of 3055 students (60.0 %). Response rate was higher among women (63.2%) than among men (49.5%) but did not vary significantly between schools (55.9 – 61.9%). The study was planned and performed by teachers in communication skills at the four universities and was registered at the National Board for Social Sciences. The study was anonymous; did not involve any kind of experiment and did not ask for sensitive personal information. Ethical approval was therefore not required according to national rules.

### Measures

We decided to use the CSAS because it addresses teaching and learning of communication skills most specifically and because it is the tool that has been most widely used and validated. The CSAS was translated into Norwegian by two independent researchers, one of them with a bachelor degree in English language. The Norwegian version was then retranslated into English by another researcher who had not seen the original version, and adjustments in the Norwegian version were made until the re-translation yielded a version similar to the original CSAS. In a pilot study the Norwegian version was tested on a sample of 78 final year Norwegian medical students, with no one reporting difficulties in understanding the questions or filling in the form (Gude, T., personal communication). The CSAS contains 26 statements concerning attitudes towards learning communication skills. Thirteen statements are positively worded (e.g.: "In order to be a good doctor I must have good communication skills" – item 1) and thirteen negatively (e.g.: "I don't need good communication skills to be a doctor" – item 19). Each statement is followed by five boxes in a Likert-like consecutive order, named "Strongly disagree", "Disagree", "Neutral", "Agree" and "Strongly agree" and is numbered from 1 to 5 respectively. The informant is asked to check one box only. Negative and positive statements are presented in a haphazard order. Each item was scored from 1 to 5 according to the box that had been checked in the questionnaires. Before analyzing the data we reversed the scores for the 13 negative items in order to obtain the same direction of scores for both negative and positive items; i.e. a higher score represents more positive attitudes for all items. The original questionnaire is presented in [5] and the Norwegian version can be obtained from the corresponding author.

### Statistical analyses

Principal component analysis (PCA), reliability tests, tests for skewness and correlation analysis (Spearman's rho) were performed by using SPSS (ver. 12.1).

## Results

### Principal component analysis and tests for skewness and reliability

**Figure 1:**
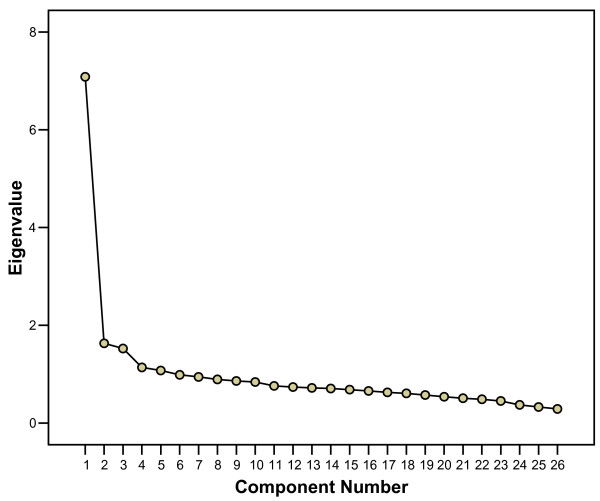
Scree Plot with Eigenvalues for each of the 26 components.

Kaiser-Meyer-Olkin measure of sampling adequacy was 0.928 and Bartlett's test of sphericity showed a significance of < 0.001, both suggesting that a principal component analysis (PCA) is feasible. The PCA with direct oblimin rotation of the scores from the 26 items in the questionnaire gave five factors with initial Eigenvalues >1 which explained 47.9% of the variance (Table 1). This table and the Scree plot (Figure 1) suggested that the CSAS mainly tests one factor explaining 27.2% of the variance. In addition, the Scree plot displayed a levelling-out from factor 4. We therefore included two additional factors, explaining 6.3% and 5.9% of the variance respectively. In selecting items to describe each of the three factors we chose the items that loaded more than 0.4 on one factor and at least 0.10 lower on any of the other two factors. The pattern matrix with loadings after rotation is shown in Table 2 and a description of the wording of the items and measures of internal reliability and skewness for each factor is shown in Table 3.

**Table 1:** Principal component analysis

	Initial Eigenvalues		
Component	Total	% of Variance	Cumulative %

1	7.084	27.245	27.245
2	1.630	6.269	33.514
3	1.524	5.862	39.376
4	1.138	4.376	43.753
5	1.074	4.131	47.883

Total variance explained by each of the five components with Eigenvalues > 1

Extraction method: Principal Component Analysis

**Table 2:** Pattern Matrix with loadings for each item on each of the three factors

	Component		
	1	2	3

Item 1	0.062	0.498	0.063
Item 2*	0.510	-0.008	-0.044
Item 3*	-0.063	0.564	0.000
Item 4	0.146	0.460	0.285
Item 5	0.280	0.153	0.590
Item 6*	0.434	0.234	-0.003
Item 7	0.708	-0.075	0.245
Item 8*	0.591	-0.014	-0.070
Item 9	0.312	0.044	0.636
Item 10	0.551	0.073	0.416
Item 11*	0.574	0.075	-0.142
Item 12	0.676	-0.086	0.271
Item 13*	0.549	-0.152	-0.109
Item 14	0.151	0.159	0.705
Item 15*	0.274	0.185	-0.251
Item 16	0.020	0.215	0.557
Item 17*	0.276	0.193	-0.290
Item 18	0.472	0.024	0.191
Item 19*	-0.046	0.582	0.076
Item 20*	0.072	0.298	-0.220
Item 21	0.693	-0.018	0.279
Item 22*	-0.128	0.708	0.033
Item 23	0.185	0.340	0.313
Item 24*	0.658	0.074	-0.023
Item 25	0.482	0.100	0.296
Item 26*	0.548	0.143	-0.014

Extraction method: Principal component Analysis

Rotation Method: Oblimin with Kaiser Normalization

Items in factor are underlined

* Item is negative and the score has been reversed before analysis

**Table 3:** Items included in each of the three factors

Factor 1 – "LEARNING" 13 items – Cronbach's α = 0.861 Skewness = -1.064
2 I can't see the point in learning communication skills *
6 I haven't got time to learn communication skills *
7 Learning communication skills is interesting
8 I can't be bothered to turn up to sessions on communication skills *
10 Learning communication skills has improved my ability to communicate with patients
11 Communication skills teaching states the obvious and then complicates it *
12 Learning communication skills is fun
13 Learning communication skills is too easy *
18 When applying for medicine, I thought it was a really good idea to learn communication skills
21 I think it's really useful learning communication skills on the medical degree
24 I find it difficult to take communication skills learning seriously *
25 Learning communication skills is important because my ability to communicate is a lifelong skill
26 Communication skills learning should be left to psychology students, not medical students *

Factor 2 – "IMPORTANCE" 5 items – Cronbach's α = 0.532 Skewness = -0.581
1 In order to be a good doctor I must have good communication skills
3 Nobody is going to fail their medical degree for having poor communication skills *
4 Developing my communication skills is just as important as developing my knowledge of medicine
19 I don't need good communication skills to be a doctor *
22 My ability to pass exams will get me through medical school rather than my ability to communicate *

Factor 3 – "RESPECTING" 4 items – Cronbach's α = 0.775 Skewness = -0.404
5 Learning communication skills has helped or will help me respect patients
9 Learning communication skills has helped or will help facilitate my team-working skills
14 Learning communication skills has helped or will help me respect my colleagues
16 Learning communication skills has helped or will help me recognise patients' rights regarding confidentiality and informed consent

Wording of items and measures of internal reliability and skewness for each factor

* Item is negative and the score is reversed

• Factor 1 comprises six positively worded items (7, 10, 12, 18, 21, 25) and seven negatively worded items (2, 6, 8, 11, 13, 24, 26). We have labelled this factor "Learning" because all items describe attitudes towards learning communication skills and all except one contain the word "learning". We suggest that this factor mainly tests students' feelings as regards the way communication skills are taught, and that this factor reflects mainly affective components of attitudes.

• Factor 2 has two positively worded (1, 4) and three negatively worded (3, 19, 22) items. We have labelled this factor "Importance" because most of the items refer to the students' perceptions of the importance of having good communication skills in order to pass exams or to become a good doctor. This factor tests students' attitudes towards relatively fundamental beliefs regarding communication skills and we think that this factor mainly reflects basic cognitive attitudes and values.

• Factor 3 has four positive items (5, 9, 14, 16) and all items claim that communication skills may be helpful for the students in order to respect patients' rights and to collaborate with colleagues and other health professionals. We have labelled this factor "Respecting" because all items state that communication skills will be helpful for the student in order to respect patients or colleagues.

Four items (15, 17, 20 and 23) were excluded because they had low and/or mixed loadings. These items deal with various topics that point in different directions.

Earlier studies of the CSAS have found that it measures two components; positive attitudes (PAS) and negative attitudes (NAS) [5,8]. One negative and 12 positive items were assigned to PAS (the score on the negative item being reversed), and one positive and 12 negative items were assigned to NAS (the score on the positive item being reversed).

### Correlation analysis

A bivariate intercorrelation matrix between the scores for all items in the CSAS showed positive correlations at a 0.05-level or higher (Spearman's rho mainly between 0.2 and 0.4) for all pairs of items. As can be seen from Table 4, most of the factors from the PCA correlate as well. However, the factor "Importance" stands out because it correlates considerably less with all the other factors.

**Table 4:** Inter-correlations between factors.

	Learning	Importance	Respecting	PAS	NAS
Learning	1.000	0.355**	0.583**	0.809**	-0.764**
Importance	0.355**	1.000	0.342**	0.532**	-0.531**
Respecting	0.583**	0.342**	1.000	0.831**	-0.396**
PAS	0.809**	0.532**	0.831**	1.000	-0.555**
NAS	-0.764**	-0.531**	-0.396**	-0.555**	1.000

PAS and NAS have been calculated according to Rees and Sheard [5].

** Correlation is significant at the 0.01 level (2-tailed)

## Discussion

Our main finding is that the CSAS may be used in order to distinguish between two different components of attitudes, namely affective ("Learning") and cognitive ("Important"). This is important because affective attitudes are easily influenced by experience while cognitive attitudes are more basic and stable. Negative affective attitudes towards learning communication skills may signal that students perceive the way skills are taught negatively, but does not necessarily mean negative attitudes towards the benefit of using such skills when seeing patients.

The difference in the outcome of the PCA in our material as compared to that of the two earlier reports may partly be due to culturally determined differences in opinions about the questions in the CSAS as well as the translation from English to Norwegian, even if translation procedure followed accepted guidelines. However, we have used the same statistical procedures as reported by Rees et al., and the computed scores for PAS and NAS in our sample are very close to theirs. In our opinion there are two main reasons for the difference. Firstly, we think that the Scree plot and the pattern of loadings indicate that a three-factor solution is more feasible than a two-factor. Secondly, we followed statistical procedures recommended by Miller et. al. [18] and selected items to be included in a factor only when loading on only one factor and low on all the others, whereas Rees et al. and Cleland et al. included all items. We believe that the two factors used by these authors describe the structure of the questionnaire (distinguishing between positive and negative items) more than the content of the items.

Our three factors do inter-correlate, and so do PAS and NAS in the earlier reports as well as in our material. We believe that the reason for this is the fact that the scores for all items do correlate to some degree. However, the correlations where the factor "Importance" is involved are much weaker than with the other factors. The correlation between the factor "Importance" and PAS/NAS is at the same level as the correlation between PAS and NAS themselves. The correlation between each of the factors "Learning" and "Respecting" on the one hand and PAS on the other hand, are much stronger (see Table 4). We think that these findings support the use of the factor "Importance" as a separate entity measuring basic cognitive attitudes and values, as distinct from "Learning" (and PAS and NAS) which mainly measure students' feelings towards the way communication skills are taught in medical school; i.e. affective attitudes.

Our response rate of 60.0% is lower than that of Rees and Sheard and that of Cleland et al. (83.8% and 86.2%). One reason for this difference may be that they handed out the questionnaires directly to the students as they attended lectures and workshops, while we used mail.

The strengths of our study are the large number of participants and its nation-wide and multi-centre cover, involving different schools and teachers with different medical specialities in collaboration on designing and performing the study and discussing the findings and the text of the final report. The weaknesses are that our factors 2 and 3 explain relatively small percentages of the variance in comparison with factor 1, that internal validity is only moderate for the items in factor 2 and that two of our factors inter-correlate.

Statistical procedures can be uncertain. Likert scales do not yield interval data strictly speaking and scores may be heavily skewed [19]. All the three factors in our analysis were negatively skewed. We have, however, tried to combat this by using non-parametric methods in the same way as other authors.

## Conclusion

Medical students' attitudes towards learning communication skills may be more complex than previously described. Our findings indicate that the two factors "Learning" and "Importance" in the CSAS may be applicable for testing affective and cognitive components of students' attitudes separately. We suggest that this may be useful for monitoring attitudinal change among students during medical school as well as making comparisons between different medical schools, making it possible to improve and refine curricula and teaching methods in communication skills.

## Competing interests

The author(s) declare that they have no competing interests.

## Authors' contributions

All authors participated in planning and designing the study, gave critical comments to the draft manuscript and approved of the final version of the manuscript. TA and TG made the statistical analyses. TA wrote the manuscript.

## Pre-publication history

The pre-publication history for this paper can be accessed here:


